# 3p22.1p21.31 microdeletion identifies *CCK* as Asperger syndrome candidate gene and shows the way for therapeutic strategies in chromosome imbalances

**DOI:** 10.1186/s13039-015-0185-9

**Published:** 2015-10-31

**Authors:** Ivan Y. Iourov, Svetlana G. Vorsanova, Victoria Y. Voinova, Yuri B. Yurov

**Affiliations:** Mental Health Research Center, 117152 Moscow, Russia; Separated Structural Unit “Clinical Research Institute of Pediatrics”, Russian National Research Medical University, Ministry of Health of Russian Federation, 125412 Moscow, Russia; Department of Medical Genetics, Russian Medical Academy of Postgraduate Education, Moscow, 123995 Russia; Moscow State University of Psychology and Education, Moscow, 127051 Russia

**Keywords:** Asperger syndrome, Autism, Bioinformatics, Chromosome abnormality, Therapeutic strategy, Candidate gene, Zinc

## Abstract

**Background:**

In contrast to other autism spectrum disorders, chromosome abnormalities are rare in Asperger syndrome (AS) or high-functioning autism. Consequently, AS was occasionally subjected to classical positional cloning. Here, we report on a case of AS associated with a deletion of the short arm of chromosome 3. Further *in silico* analysis has identified a candidate gene for AS and has suggested a therapeutic strategy for manifestations of the chromosome rearrangement.

**Results:**

Using array comparative genomic hybridization, an interstitial deletion of 3p22.1p21.31 (~2.5 Mb in size) in a child with Asperger’s syndrome, seborrheic dermatitis and chronic pancreatitis was detected. Original bioinformatic approach to the prioritization of candidate genes/processes identified *CCK* (cholecystokinin) as a candidate gene for AS. In addition to processes associated with deleted genes, bioinformatic analysis of *CCK* gene interactome indicated that zinc deficiency might be a pathogenic mechanism in this case. This suggestion was supported by plasma zinc concentration measurements. The increase of zinc intake produced a rise in zinc plasma concentration and the improvement in the patient’s condition.

**Conclusions:**

Our study supported previous linkage findings and had suggested a new candidate gene in AS. Moreover, bioinformatic analysis identified the pathogenic mechanism, which was used to propose a therapeutic strategy for manifestations of the deletion. The relative success of this strategy allows speculating that therapeutic or dietary normalization of metabolic processes altered by a chromosome imbalance or genomic copy number variations may be a way for treating at least a small proportion of cases of these presumably incurable genetic conditions.

## Background

Asperger syndrome (AS) is a neurodevelopmental condition clinically and genetically linked to autism spectrum disorders (ASD), manifesting as appreciable difficulties in social interaction and nonverbal communication with restricted and repetitive patterns of behavior and interests [1, 2]. Although chromosomal abnormalities and genomic copy number variations are common in ASD [3–9], genome variations are occasionally reported in individuals with AS [10, 11]. Such rarity of chromosomal rearrangements hinders the application of classical positional cloning [12]. In the available literature, only exceptional cases of individual chromosome imbalances (i.e. deletions, duplications, translocations, supernumerary marker chromosomes, and gonosomal aneuploidy) or chromosomal syndromes (i.e. 3q26.33-3q27.2 microdeletion and Klinefelter syndromes) have been associated with AS [10, 11, 13–20]. Since occasional chromosome studies and genome-wide analyses of AS yielded conflicting results [21–23], the search for candidate genes of this ASD remains to be pursued.

Recently, we have used several array comparative genomic hybridization (CGH) platforms for studying Russian cohort of children with ASD and/or intellectual disability (described previously [4, 24–26]). Additionally, an original bioinformatic technology [27, 28] was used to determine functional consequences of genomic rearrangements and pathogenetic mechanisms. In a case of AS, a deletion of the short arm of chromosome 3 was detected.

## Results and discussion

**Fig. 1:**
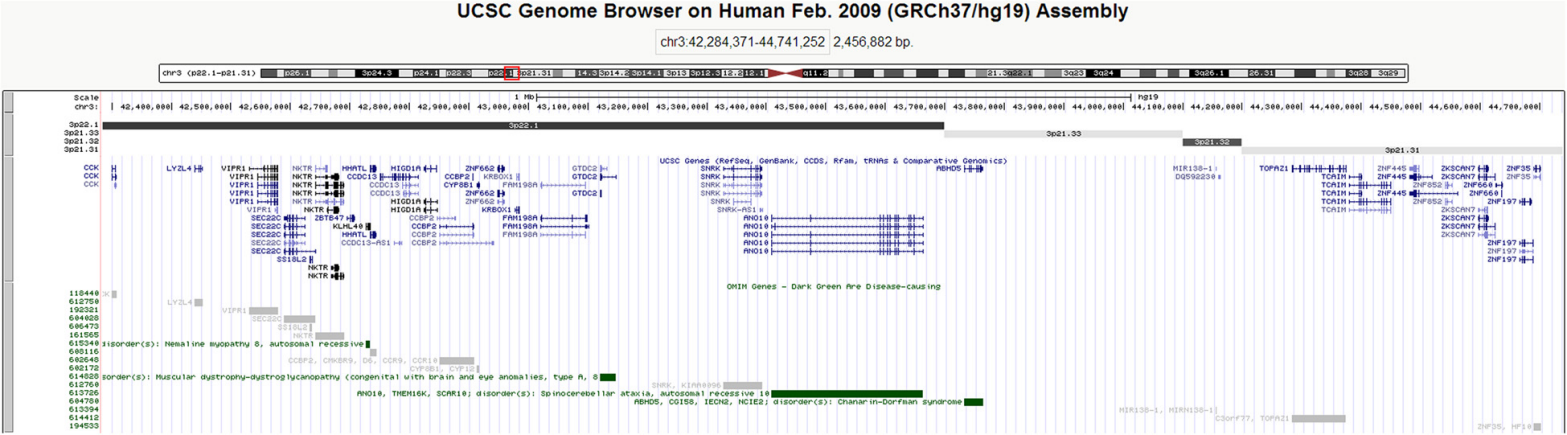
Schematic overview of the deletion of the short arm of chromosome 3 (3p22.1p21.31) depicted using UCSC Genome Browser (Human Feb. 2009 (GRCh37/hg19 assembly), http://genome-euro.ucsc.edu/index.html) showing OMIM (Online Mendelian Inheritance in Man) genes (genes associated with OMIM disorders are shown in green)

**Fig. 2:**
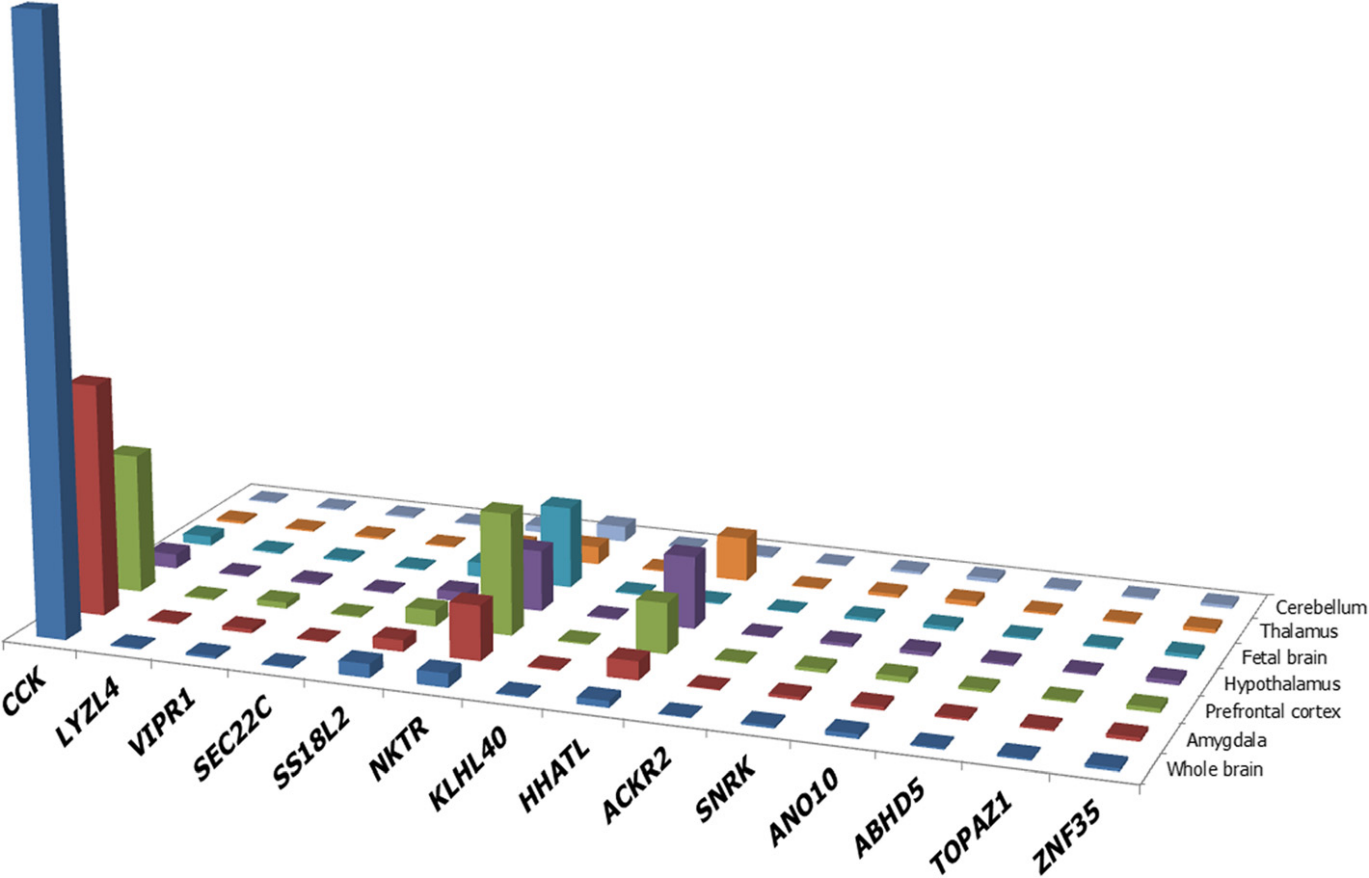
Gene expression profiles of deleted genes in brain areas known to be involved in ASD pathophysiology (according to [29]); data was retrieved from BioGPS (http://biogps.org [30])

An interstitial deletion of 3p22.1p21.31 (2.456 Mb in size) in a child with Asperger’s syndrome, seborrheic dermatitis and chronic pancreatitis was detected (Fig. 1). According to the available literature, these chromosomal regions were never deleted in in children with ASD. However, previous linkage analyses have mapped AS to 3p21-3p24 [21]. The deletion encompassed 4 genes associated with autosomal recessive diseases, none of which was observed in the index case. To evaluate functional consequences of the gene loss, we have further analyzed these genes using an original bioinformatic methodology [28]. The selection of brain areas for gene prioritization through gene expression profiling was made according to Amaral et al. [29], who had summarized brain regions affected in ASD. *In silico* gene expression profiling of deleted genes using BioGPS [30] have indicated that *CCK* is the most likely candidate gene for AS. Although *NKTR* and *HHATL* have also indicated an increased expression in brain areas of interest, *CCK* has shown the highest expression (prioritization score) (Fig. 2). *CCK* encodes cholecystokinin, a brain/gut peptide inducing the pancreatic enzyme release and gallbladder contraction. In brain functioning, *CCK* role remains unclear. However, it is suggested to be involved in a variety of neuropsychiatric disorders [31] and normal or pathological eating behavior [32, 33]. Genomic copy number variations of *CCK* have never been associated with ASD.

Currently, analyses of chromosome abnormalities in AS have suggested several candidate genes: *NIPA1* [11], *MINK1* and *MINK2* [14], *ZFP536* [18], *LFNG* [19]. Genome-wide association and linkage studies have not found a clear signal at a gene for AS [21–23]. Therefore, one can suggest that a discovery of a new AS candidate gene is a valuable contribution to ASD research.

**Fig. 3:**
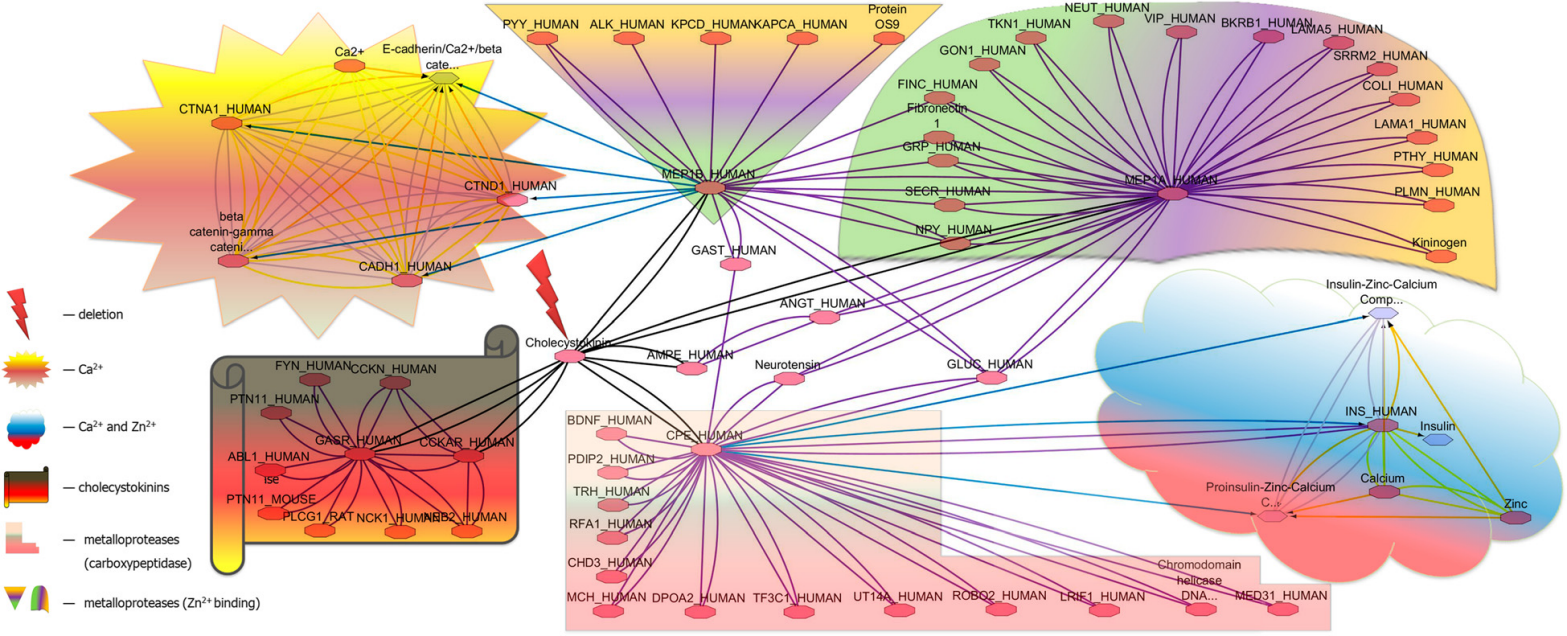
*CCK* interctome (protein interaction network). Using irregular geometric shapes/cartoons, interctome parts related to different pathways and/or molecular functions are depicted: Ca^*2+*^-calcium metabolism; Ca^*2+*^ and Zn^*2+*^-(pro-)insulin-zinc-calcium complexes; cholecystokinins-interactome part related to cholecystokinin receptor pathway (i.e. *CCK-*regulated food intake); metalloproteases (carboxypeptidase)-interactome part related to the pathway of biosynthesis of peptide hormones and neurotransmitters (including insulin) being a likely link between *CCK-*regulated food intake pathways and zinc metabolism; metalloproteases (Zn^*2+*^ binding)-interactome parts related to MEP1A- and MEP1B-mediated zinc ion binding, which interact with *CCK*. The interactome was processed by Cytoscape software (Version: 3.1.1) [48]

To gain further insights into the *CCK*-mediated mechanisms of the disease and confirm its involvement in ASD, we have addressed *CCK* interactome (protein interaction network) (Fig. 3). The analysis has revealed a likely mechanism for clinical manifestations. Firstly, alterations to *CCK* are more likely to result in abnormal eating behavior and pancreatic problem [34]. The latter has been manifested as pancreatitis in the index patient. Additionally, we have hypothesized that an alteration to key element of this protein interaction network/pathway (Fig. 3) is likely to result not only in general malabsorption, but also in reduced absorption of calcium and zinc. This hypothesis was based on the fact that interactome parts related to calcium/ zinc metabolism (i.e. formation of (pro-)insulin-zinc-calcium complexes and MEP1A- and MEP1B-mediated zinc ion binding) were likely to be “disconnected” from cholecystokinin receptor pathway or CCK*-*mediated food intake because of “*CCK* removal”. Biochemically, low zinc plasma zinc concentration was revealed. Calcium concentrations were normal. It is noteworthy, that zinc deficiency is constantly reported to feature ASD [35–37]. Therefore, it was not surprising that AS and other phenotypic manifestations of the deletions were etiologically related to zinc malabsorption/deficiency. Consequently, increasing the zinc intake produced a rise in zinc plasma concentration coupled with the improvement in the patient’s condition (for more details see Case report). It is to note that such psychopharmacological interventions are not common in ASD [38, 39].

Combining molecular cytogenetic whole genome scan (i.e. array CGH or chromosomal microarray analysis) with *in silico* approaches to uncover molecular and cellular mechanisms of genomic diseases in a personalized manner has been proposed earlier [40–42]. In this study, the potential of this emerging technology has been increased inasmuch as data on a chromosome abnormality was used not only for diagnostic issues, but also for developing a therapeutic strategy. More importantly, since chromosome syndromes and genomic disorders are generally considered incurable, new perspectives on treating at least a small proportion of cases associated with single molecular defects altered by genomic copy number variations/chromosomal imbalances appear to be highly attractive. Therefore, in addition to all the benefits, which may derive from clinical and research applications of chromosomal microarray-based technologies (given in details in [43]), a new one can be added to the list.

## Conclusion

In conclusion, molecular cytogenetic and *in silico* analyses of a deletion of the short arm of chromosome 3 in a patient with AS, seborrheic dermatitis, and chronic pancreatitis have supported previous linkage findings and have implicated *CCK* as a new candidate gene for AS. Furthermore, we were able to propose the pathogenic mechanism, which allowed to develop a more-or-less successful therapeutic strategy. Accordingly, we speculate that either therapeutic or dietary normalization of metabolic processes produced by chromosome imbalances or genomic copy number variations might be a way for treating at least a small proportion of individuals suffering from these presumably incurable genetic conditions.

## Methods

### Case report

The proband is the only child of unrelated parents. His father meets DSM-IV criteria for social phobia. Family history is otherwise negative for ASD but two maternal uncles had a history of alcohol abuse. The boy was born by cesarean delivery at 39 weeks after a pregnancy marked by bleeding events during the first trimester. Neonatal measurements were within normal limits. His birth weight was 3200 g. His length was 52 cm. The boy had a prolonged period of neonatal jaundice and high muscle tone. Early motor milestones were delayed. He had good head control at age of 3 months; he rolled over at 6 months, sat up at 8 months, began to stand at 9 months. He started walking unsteadily without support at 15 months and had poor motor coordination until 5 years of age. He spoke his first words at 11 months, used sentences by 30 months. He knew the letters at this age. At the age of 4 years, he learned to read all by himself, had excellent memory for poems and was able to perform easy calculations. However he was often awkward in social situations and showed no interest to other children usually playing at a distance from others. At the age of 8 years, physical examination showed no minor abnormalities except large ears, flattened midface and ocular hypotelorism. The boy had low weight 21.5 kg (10^th^ percentile) and poor subcutaneous fat. His height was 128 cm, and head circumference was 53 cm (average). He suffered from seborrheic dermatitis and frequent respiratory infections. Biliary dyskinesia and chronic pancreatitis were diagnosed by a gastroenterologist. Special diet and pancreatic enzyme supplements were inefficient. The patient also had a mitral valve regurgitation, nephroptosis and myopia. He was quite clumsy and had motor stereotypic hand movements referred to shaking hands during agitation. His social interaction was impaired. Direct eye gaze feedback was rare. Limited use of gestures and the phobia of communicating with people during mealtimes were noted. Boy’s interests were focused on computer and he was making a substantial progress in this activity. The proband showed an IQ within the normal range (110) and fulfilled DSM-IV criteria for Asperger syndrome. Cytogenetic analyses showed normal karyotypes in the index patient and his parents. Biochemical studies showed a reduced plasma zinc concentration (6.5 μmol/L) and normal serum calcium concentration (2.4 mmol/L). Accordingly, zinc gluconate *per os* for the normalization of the plasma concentration were proposed. Three months after its administration, boy’s weight increased to 23.1 kg and seborrheic dermatitis disappeared. Communication became more intense and stereotyped movements were rarer. The patient did not experienced further episodes of pancreatitis. Plasma zinc concentration reached normal levels (14.3 μmol/L).

### Molecular cytogenetics (Array CGH)

Array CGH was performed using BAC and oligonucleotide array CGH: Human BAC Array-System, Perkin Elmer and NimbleGen 135 K whole genome tiling array, respectively. BAC-array CGH was performed using customized Constitutional Chip®4.0 (Human BAC Array-System, Perkin Elmer, USA) as described earlier [25, 44]. The resolution of the BAC-array was estimated as 0.3–05 Mb. Oligonucleotide array CGH was performed using NimbleGen 135 K whole genome tiling array as previously described [45, 46]. Sample was labeled using Cy3-dUTP whereas reference DNA was labeled by Cy5-dUTP. Hybridization was done according to the manufacturer’s instructions (NimbleGen Arrays User’s Guide CGH and CGH/LOH Arrays v9.1, Roche NimbleGen, Madison, WI, USA). Scanning and image acquisition has been processed in the same way as for BAC-Perkin Elmer Array [25, 45].

### Bioinformatics

Genomic, epigenomic, proteomic and metabolomic data was analyzed as described previously [27, 28, 47]. Each deleted gene was addressed using clinical, genomic (browsers and gene ontology databases), epigenetic (gene expression), proteomic, interactomic (database + software) and metabolomic databases. Interactomic data was visualized and processed using Cytoscape software (Version: 3.1.1) [48]. The technology of prioritization of candidate genes/processes was originally described in details in [28].

## Abbreviations

AS: Asperger syndrome
ASD: Autism spectrum disorders
CGH: Comparative genomic hybridization
